# Supplementary material for: Niche-related processes explain phylogenetic structure of acoustic bird communities in Mexico
Source: PeerJ. 2025 Jan 8;13:e18412. doi: 10.7717/peerj.18412 (PMC11724652; doi:10.7717/peerj.18412)
Supplement: Supplemental Information 1 — W = Shapiro-Wilk test statistic, P = probability value. [file peerj-13-18412-s001.docx]

**Table S1.** Shapiro-Wilk normality test values for phylogenetic indices (PD, NRI, NTI), acoustic indices (BI and ACI), vegetation structure, and excess attenuation. W = Shapiro-Wilk test statistic, P = probability value.

|  | W | ***P*** |
| --- | --- | --- |
| PD | 0.95 | 0.5183 |
| NRI | 0.74 | 0.0007 |
| NTI | 0.95 | 0.4584 |
| BI | 0.95 | 0.5337 |
| ACI | 0.95 | 0.5533 |
| Vegetation structure | 0.94 | 0.3418 |
| Excess attenuation | 0.97 | 0.7965 |
